# An interpretable artificial intelligence model based on CT for prognosis of intracerebral hemorrhage: a multicenter study

**DOI:** 10.1186/s12880-024-01352-y

**Published:** 2024-07-09

**Authors:** Hao Zhang, Yun-Feng Yang, Xue-Lin Song, Hai-Jian Hu, Yuan-Yuan Yang, Xia Zhu, Chao Yang

**Affiliations:** 1 Department of Radiology, The First Affiliated Hospital of Dalian Medical University, Dalian, 116000 Liaoning China; 2 Laboratory for Medical Imaging Informatics, Shanghai Institute of Technical Physics, Chinese Academy of Sciences, Shanghai, 200083 China; 3 Laboratory for Medical Imaging Informatics, University of Chinese Academy of Sciences, Beijing, 100049 China; 4 Department of Radiology, The Second Affiliated Hospital of Dalian Medical University, Dalian, 116027 Liaoning China; 5 Department of Hemato-oncology, The First Hospital of Changsha, Changsha, 410005 Hunan China; 6 Department of Gynecology, Hunan Provincial Maternal and Child Health Care Hospital, Changsha, 410028 Hunan China

**Keywords:** Cerebral hemorrhage, Radiomics, Deep learning, Computed tomography, Interpretable model

## Abstract

**Objectives:**

To develop and validate a novel interpretable artificial intelligence (AI) model that integrates radiomic features, deep learning features, and imaging features at multiple semantic levels to predict the prognosis of intracerebral hemorrhage (ICH) patients at 6 months post-onset.

**Materials and methods:**

Retrospectively enrolled 222 patients with ICH for Non-contrast Computed Tomography (NCCT) images and clinical data, who were divided into a training cohort (*n* = 186, medical center 1) and an external testing cohort (*n* = 36, medical center 2). Following image preprocessing, the entire hematoma region was segmented by two radiologists as the volume of interest (VOI). Pyradiomics algorithm library was utilized to extract 1762 radiomics features, while a deep convolutional neural network (EfficientnetV2-L) was employed to extract 1000 deep learning features. Additionally, radiologists evaluated imaging features. Based on the three different modalities of features mentioned above, the Random Forest (RF) model was trained, resulting in three models (Radiomics Model, Radiomics-Clinical Model, and DL-Radiomics-Clinical Model). The performance and clinical utility of the models were assessed using the Area Under the Receiver Operating Characteristic Curve (AUC), calibration curve, and Decision Curve Analysis (DCA), with AUC compared using the DeLong test. Furthermore, this study employs three methods, Shapley Additive Explanations (SHAP), Grad-CAM, and Guided Grad-CAM, to conduct a multidimensional interpretability analysis of model decisions.

**Results:**

The Radiomics-Clinical Model and DL-Radiomics-Clinical Model exhibited relatively good predictive performance, with an AUC of 0.86 [95% Confidence Intervals (CI): 0.71, 0.95; *P* < 0.01] and 0.89 (95% CI: 0.74, 0.97; *P* < 0.01), respectively, in the external testing cohort.

**Conclusion:**

The multimodal explainable AI model proposed in this study can accurately predict the prognosis of ICH. Interpretability methods such as SHAP, Grad-CAM, and Guided Grad-Cam partially address the interpretability limitations of AI models. Integrating multimodal imaging features can effectively improve the performance of the model.

**Clinical relevance statement:**

Predicting the prognosis of patients with ICH is a key objective in emergency care. Accurate and efficient prognostic tools can effectively prevent, manage, and monitor adverse events in ICH patients, maximizing treatment outcomes.

**Supplementary Information:**

The online version contains supplementary material available at 10.1186/s12880-024-01352-y.

## Key points

•A multimodal AI model combining imaging features, deep learning features, and radiomics features at three different semantic levels outperforms traditional radiomics models;

•This study proposes an interpretable deep learning radiomics model that enables the model to maintain a certain level of interpretability while meeting high-performance requirements;

**Supplementary Information:**

The online version contains supplementary material available at 10.1186/s12880-024-01352-y.

## Introduction

Intracerebral hemorrhage (ICH) is a life-threatening condition and a subtype of stroke, accounting for approximately 10–15% of all strokes. It continues to carry a high mortality and disability rate, and improving prognosis requires proactive decision-making and management of patients in the early stages of the disease [1–4]. Over the past decade, ICH-related research has significantly increased, identifying numerous factors associated with acute ICH prognosis. Models predicting patient outcomes and functional prognosis based on clinical factors such as Glasgow Coma Scale (GCS) score, NIH Stroke Scale (NIHSS) score, patient age, blood pressure levels, hematoma volume, and hematoma location during hospitalization have been widely developed [5]. Non-contrast Computed Tomography (NCCT) is the preferred imaging modality for the clinical diagnosis of acute cerebral hemorrhage. Many imaging features displayed on NCCT have been proven to aid in predicting hematoma expansion and prognosis, such as the black hole, density grading, shape grading, blend sign, island sign, and swirl sign, among others [6–11]. Additionally, the “spot sign” on computed tomography angiography (CTA) images has been considered an effective predictor of poor prognosis but is limited by the fact that early CTA is not readily available in many medical centers [12, 13]. Research indicates that the majority of patients who survive after a cerebral hemorrhage are left with disabilities and are at risk of recurrent stroke, decreased cognitive abilities, and systemic vascular diseases [14]. Early prediction of prognosis in ICH patients is beneficial for clinicians in providing timely treatment interventions and improving patient outcomes [15].

Unlike traditional imaging analysis methods, radiomics involves the extraction of a large number of features from medical images for quantitative analysis. It can provide more comprehensive and objective microscopic information for disease analysis. Radiomics offers nearly infinite imaging biomarkers, which may aid in cancer detection, diagnosis, and treatment [16, 17]. In recent years, artificial intelligence (AI) algorithms have gained significant traction in medical imaging [18]. There have been several relevant studies on prognostic models for intracerebral hemorrhage [19–23], but these studies have primarily focused on extracting imaging features from NCCT or radiomic feature types, without considering deep learning features and their integration modeling among the three. However, data from different modalities often provide complementary information, and modeling with multimodal information can yield richer and more comprehensive data representations, thus improving the representational capability of the data [18, 24]. Studies have shown that with the clinical assistance of medical AI models, there has been a significant improvement in the accuracy of diagnostic decisions made by physicians [25]. Generally, complex machine learning algorithms have a higher performance ceiling than simple ones, but they often lack interpretability [26, 27]. Currently, there is limited research on the interpretability of medical AI models [27, 28]. This study proposes an interpretable artificial intelligence research framework that integrates three model interpretation methods (SHAP, Grad-CAM, and Guided Grad-CAM) to explain the models while utilizing efficient AI models for image feature extraction and prediction [29, 30].

This study integrates imaging features, radiomics features, and deep learning features to construct a multimodal interpretable model for predicting the prognosis of patients with cerebral hemorrhage. To the best of our knowledge, this is the first study to propose a joint interpretable framework combining machine learning and deep learning for predicting the prognosis of cerebral hemorrhage.

## Materials and methods

### Patient characteristics

This retrospective study was approved by the Ethics Committee of Medical Center 1 and adhered to the principles outlined in the Helsinki Declaration throughout the research process.

**Fig. 1:**
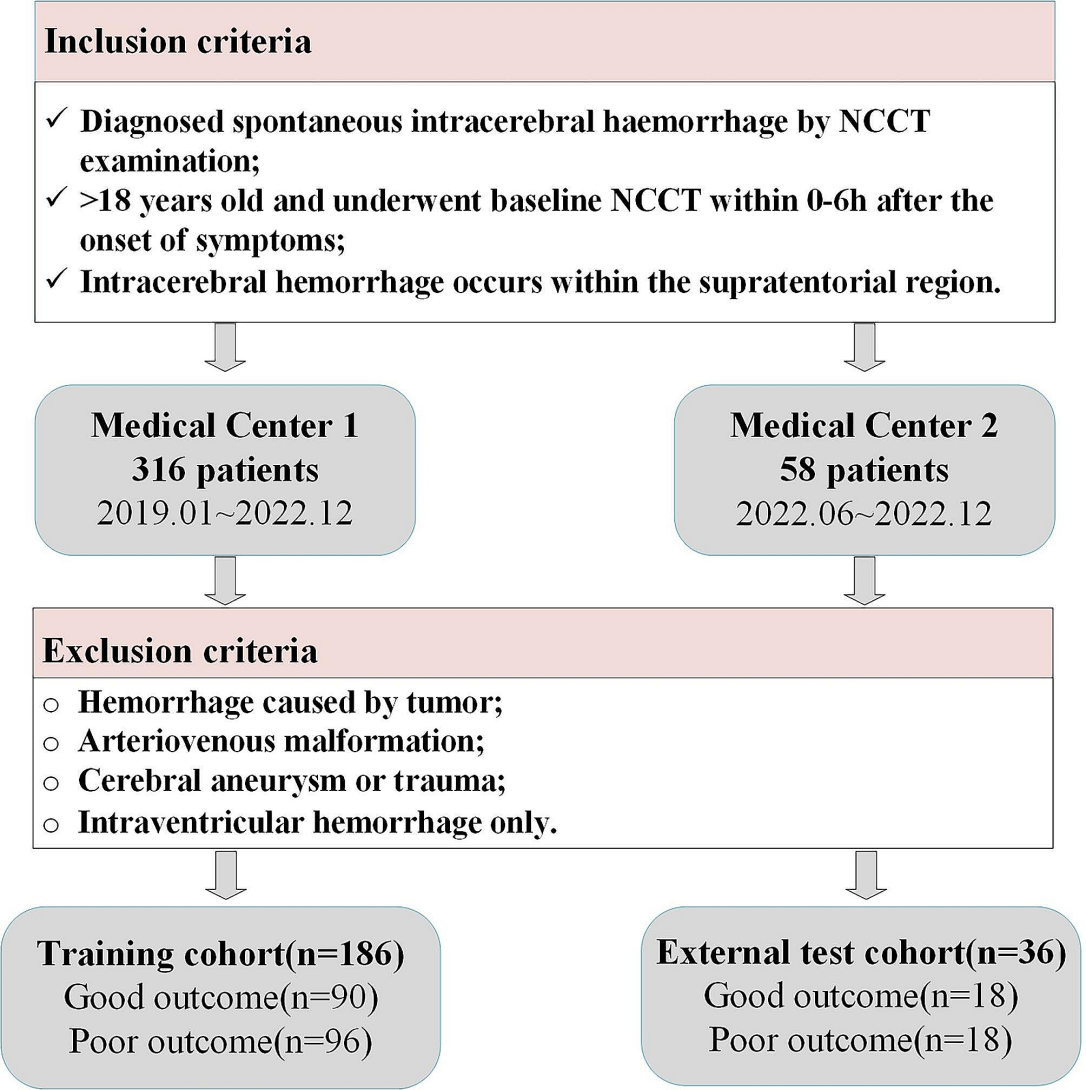
Patient enrollment flowchart

Retrospective data were collected from spontaneous cerebral hemorrhage patients who were admitted to Medical Center 1 between January 2019 and December 2022, and to Medical Center 2 between June 2022 and December 2022. Figure 1 provides the inclusion/exclusion criteria and detailed information on patient enrollment for this study, involving a total of 222 patients. The 186 samples from Medical Center 1 will be used as the training set, while data from Medical Center 2 will be combined to form a separate external testing dataset. This approach effectively prevents overfitting of the model and maximizes the reliability of the model’s performance.

### Outcome assessment

Two radiologists with over five years of experience assessed the functional outcomes of patients at six months using the modified Rankin Scale (mRS). Referring to previous studies, an mRS score of 0–2 is considered a favorable prognosis, while a score of 3–6 is considered an unfavorable prognosis [31, 32]. As shown in Fig. 1, at Medical Center 1, there are 90 cases of “Good outcome” and 96 cases of “Poor outcome”; at Medical Center 2, there are 18 cases each for “Good outcome” and “Poor outcome.” The overall label distribution is relatively balanced.

### Clinical factors and imaging features analysis

**Fig. 2:**
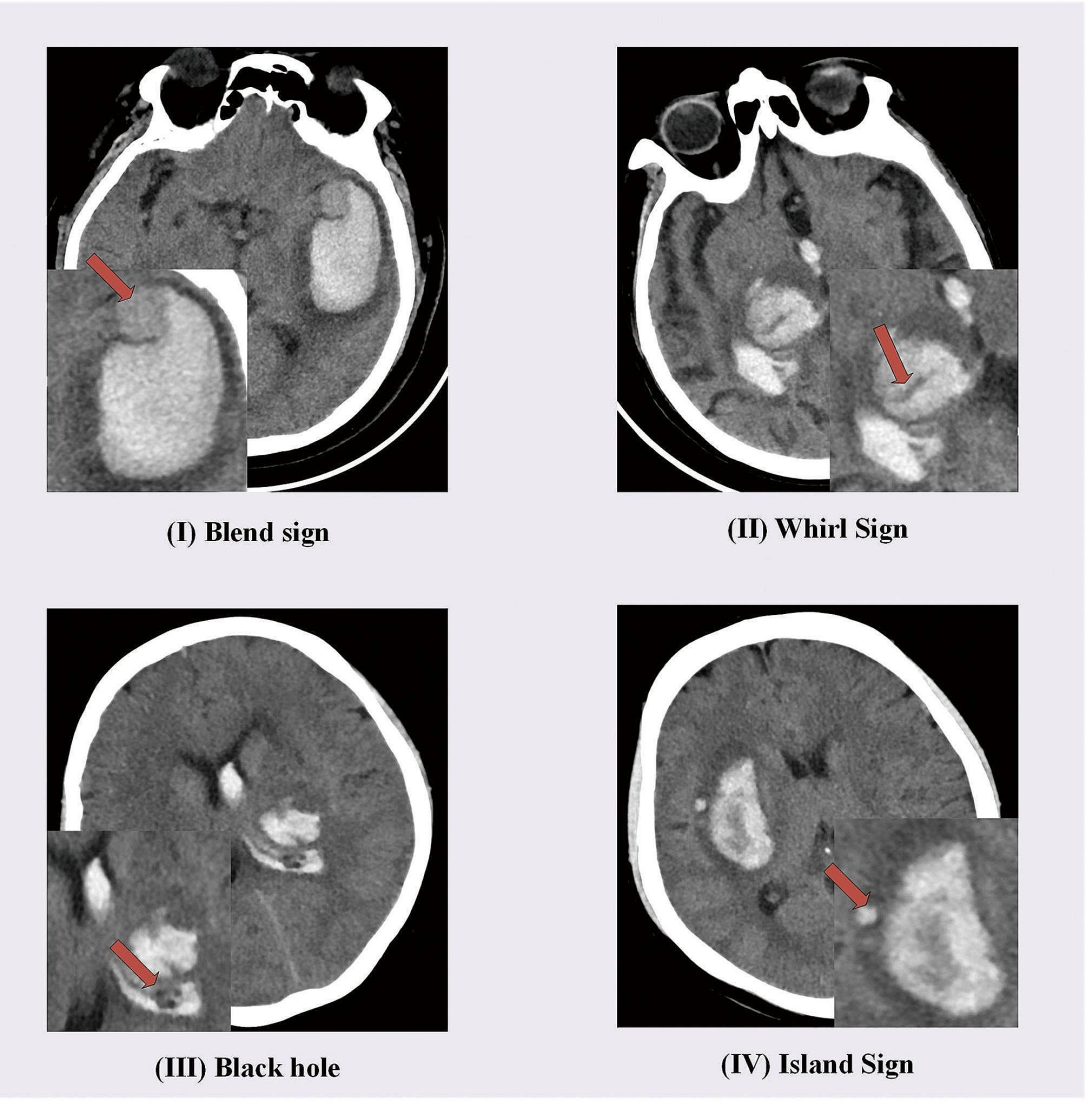
Schematic representation of some imaging features on NCCT

The clinical factors of enrolled patients, including age, gender, hypertension, and diabetes, were collected from the information systems of various hospital centers. Additionally, two radiologists with five years and seven years of experience, respectively, evaluated the radiological features that have been confirmed in previous studies [6–11] to be closely associated with the prognosis of cerebral hemorrhage. In cases of disagreement between the two radiologists, a third radiologist with 15 years of imaging experience made the final determination. Radiologists independently evaluated and analyzed NCCT images of the patients without knowledge of their outcomes. The analysis included (1) blend sign, (2) island sign, (3) swirl sign, (4) fluid level, (5) black hole, (6) density grading, (7) intraventricular hemorrhage, and (8) shape grading. These clinical factors and imaging features are all either binary, multicategory, or continuous numerical data. To standardize these data, they were numerically encoded and normalized using the Min-max normalization method, scaling all features to a range between 0 and 1. This approach helps to mitigate errors resulting from differences in scale among different features and facilitates data processing and feature fusion. Figure 2 illustrates specific manifestations of some typical radiological features of NCCT.

### CT imaging protocol

All patients participating in the study underwent NCCT scans within 6 h of the onset of stroke symptoms. The entire dataset underwent quality checks, and cases with significant artifacts were excluded. Detailed information regarding the imaging protocols and parameters can be found in Supplementary material 1.

### Research pipeline

**Fig. 3:**
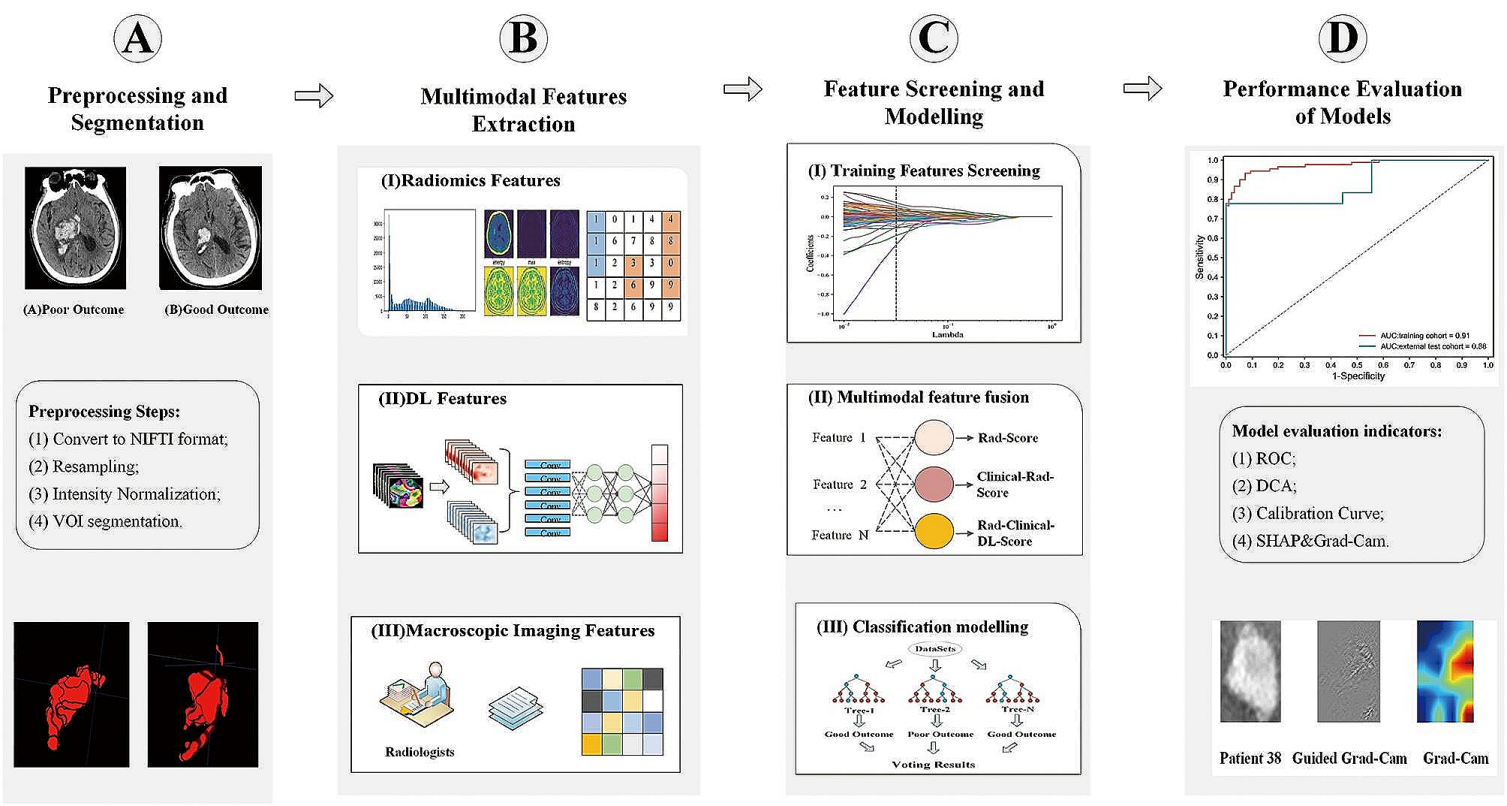
The pipeline of the study

The overall flowchart of the model consists of the following components: (A) Preprocessing and Segmentation, (B) Multimodal Features Extraction, (C) Feature Screening and Modelling, and (D) Performance Evaluation of Models. Details are illustrated in Fig. 3.

### Data pre-processing and VOI segmentation

After exporting the NCCT images of all patients from the PACS system, they were converted to the Neuroimaging Informatics Technology Initiative (NIFTI) format. The images were resampled to a uniform spatial resolution of 0.5 mm x 0.5 mm x 5 mm using the nearest neighbor interpolation algorithm from the SimpleITK algorithm library, with a slice spacing of 5 mm. The min-max normalization method was employed to normalize the intensity of all images to a range of 0 to 1, and the window width and level were uniformly adjusted. These preprocessing methods aimed to mitigate partial deviations caused by variations in imaging instrument parameters.

A radiologist (with five years of imaging diagnostic experience) initially segmented the volume of interest (VOI) along the hematoma contours in the NCCT images of all 222 patients. Subsequently, 20 cases were randomly selected from all enrolled patient images, and another radiologist (with seven years of imaging diagnostic experience) performed a second round of VOI segmentation. All image segmentation procedures were carried out using ITK-Snap software (version 4.0.1, www.itksnap.org). After completing all VOI segmentations, SimpleITK and numpy algorithm libraries were utilized to read the VOIs and calculate the hematoma volume for each patient. For detailed technical details on image preprocessing, segmentation, and feature extraction, please refer to Supplementary material 2.

### Radiomics features extraction

The Pyradiomics feature extraction library (https://pyradiomics.readthedocs.io/en/latest/) was employed to extract 1762 radiomics features from each NCCT image. The extracted features adhere to the Image Biomarker Standardization Initiative (IBSI) standard [33]. For detailed information regarding the specific categories and quantities of all radiomics features, please refer to Supplementary material 2.

### Deep learning features extraction

For all patients’ NCCT images and their corresponding VOIs, the SimpleITK library (https://simpleitk.org/) was utilized to iteratively crop all two-dimensional rectangular Regions of Interest (ROIs), retaining only the slice with the largest hematoma cross-sectional area in each patient’s NCCT image. This process resulted in 222 ROIs representing the largest lesion cross-sections, which were used as training and external test set data. This study employed an efficient deep learning model (EfficientNetV2) for model training and feature extraction. EfficientNetV2 is a lightweight and efficient model that incorporates advanced model scaling strategies, optimizing network width, depth, and resolution to achieve improved performance and higher parameter efficiency [34, 35]. In order to enhance the model’s feature extraction capability, it was initially pre-trained using the brain tumor grade classification dataset from MICCAI 2018 BRATS [36] to improve its ability to extract features from medical imaging data [37]. Subsequently, the model was fine-tuned using the 186 training set images from this study to achieve optimal performance, extracting 1000 deep learning features from the output of the final convolutional layer in the model. Further detailed information regarding the hyperparameters for the training of the EfficientNetV2 model and the MICCAI dataset can be found in Supplementary Material 2.

### Feature screening and feature fusion

To calculate the intraclass correlation coefficient (ICC) for the aforementioned 20 sets of repetitively segmented image VOIs to remove unstable features (ICC < 0.75), the radiomics feature data for these 20 patients can be found in Supplementary material 3. The remaining features underwent Z-Score standardization, and redundant features were eliminated using either Pearson or Spearman methods. Subsequently, the ElasticNet algorithm was utilized to compute the Rad-Score, while the minimal-redundancy-maximal-relevance criterion (mRMR) algorithm was applied to select the optimal radiomics features.

To further incorporate clinical factors with imaging features and perform feature selection and fusion, initial univariate logistic regression analysis was conducted to pre-select clinically significant variables with *P* < 0.05. The pre-selected clinical factors, along with the radiomics features identified in the previous step, were combined, and the fused feature Clinical-Rad-Score was computed using the ElasticNet algorithm. Simultaneously, the mRMR algorithm was employed to obtain the optimal features.

Finally, deep learning features were incorporated. Using Multi-Layer Perception (MLP), 1000 deep learning features were mapped to derive the top 10 most representative deep learning features. The optimal radiomics features obtained previously were combined with clinical factors and deep learning features, and the mRMR algorithm was applied to select ten features with the highest predictive value. Regarding the technical details of mRMR, please refer to Supplementary material 4. Subsequently, the fused feature DL-Clinical-Rad-Score was computed using ElasticNet. The computation process of DL-Clinical-Rad-Score is similar to that of Rad-Score and Clinical-Rad-Score, where regression coefficients from ElasticNet are multiplied by feature values, summed, and then added a bias coefficient. The difference lies in the input features provided to ElasticNet. For detailed technical details on feature selection, please refer to Supplementary material 2.

### Model development and evaluation

Based on the feature selection and fusion results mentioned above, this study utilized three different training feature combinations as input data for training prognostic models of different categories. Specifically, these are (1) Radiomics Model: a combination of radiomics features and the fused feature Rad-Score; (2) Radiomics-Clinical Model: radiomics features, imaging features, clinical factors, and Clinical-Rad-Score; (3) DL-Radiomics-Clinical Model: radiomics features, imaging features, clinical factors, deep learning features, and DL-Clinical-Rad-Score.

Before model training, this study employed the Synthetic Minority Oversampling Technique (SMOTE) [38] to balance the samples of different data label categories in the training set. This method can effectively improve the training efficiency and performance limits of the model. For more detailed information on the details and effects of the SMOTE technique, please refer to Supplementary material 4. All models in this study selected the RF algorithm as the classifier for the models. The RF model is known for its high accuracy and strong resistance to overfitting and is currently widely used in various machine-learning tasks [39]. Upon completion of model training, this study utilized AUC, calibration curve, and DCA to evaluate the performance and clinical applicability of the models. Additionally, the Delong test was employed to compare the AUC of different models.

To enhance the transparency of the model’s decision-making process, Grad-CAM and Guided Grad-CAM [30] were employed for visualizing deep learning models. We utilized the gradient information from the last convolutional layer of CNNs for weighted fusion, generating a class activation map that highlights important regions of the target image for classification. For technical details on Grad-CAM and Guided Grad-CAM, please refer to Supplementary material 4. For the final RF classification model, this paper utilizes the SHAP model explanation method [29] to visualize and quantify the contribution of each feature to the model’s decision-making process.

### Statistics

Statistical analysis was performed using SPSS 26.0 software (https://www.ibm.com/spss) and Python 3.9 (https://www.python.org/). Normality tests were conducted on the statistical data, with chi-square tests used for categorical data. Logistic regression was employed to analyze clinical factors and imaging features, and the results were expressed as odds ratios (OR) and 95% CI. A p-value of < 0.05 was considered indicative of statistical significance. The “sklearn. metrics”, “matplotlib,” and “sklearn. calibration” algorithm libraries were used to calculate the model’s AUC, sensitivity, and specificity and to generate calibration and decision curves. The AUC of each model was compared using the Delong test.

## Results

### Patient characteristics

Table 1 presents the results of univariate analysis of detailed imaging features and clinical factors for all enrolled samples. A total of 10 features significantly associated with the prognosis of intracerebral hemorrhage were pre-screened from the imaging features and clinical factors, including Intraventricular Hemorrhage, Blend Sign, Island Sign, Whirl Sign, Black hole, Hematoma volume, Density grading, Shape grading, as well as two composite features: Shape-Density Grading (the fusion of density grading and shape grading) and BH-WS-IS-B (the fusion of black hole, whirl sign, island sign, and blend sign). Table 2 provides the results of univariate analysis of imaging features and clinical factors for the training set and external test set, respectively.

**Table 1:** The statistical results of univariate analysis for radiological features and clinical factors of all eligible sample data

Variables	Univariate analysis	
	OR (95%CI)	*P* value
Age	0.98(0.96,1.00)	0.13
Sex	1.50(0.86,2.64)	0.16
Diabetes	0.63(0.31,1.25)	0.18
Hypertension	0.71(0.38,1.34)	0.29
Intraventricular hemorrhage	0.40(0.20,0.80)	**0.01**
Blend sign	0.23(0.10,0.54)	**< 0.01**
Island sign	0.13(0.05,0.35)	**< 0.01**
Whirl sign	0.41(0.24,0.71)	**< 0.01**
Fluid level	1.46e(-9)	1.00
Black hole	0.20(0.07,0.56)	**< 0.01**
Hematoma volume	0.96(0.94,0.98)	**< 0.01**
Density grading	0.35(0.25,0.50)	**< 0.01**
Shape grading	0.57(0.46,0.71)	**< 0.01**
Shape-Density Grading*	0.70(0.61,0.80)	**< 0.01**
BH-WS-IS-M*	0.33(0.23,0.47)	**< 0.01**

* Shape-Density Grading, the fusion of density grading and Shape grading. BH-WS-IS-M, the fusion of black hole, whirl sign, island sign and blend sign. P-values are statistically significant in boldface format. OR, Odds ratio. CI, Confidence interval

**Table 2:** The statistical results of univariate analysis of radiological features and clinical factors in the training set and external test set, respectively

Variables	Training cohort (*n* = 186)		External test cohort (*n* = 36)	
	OR (95%CI)	*P* value	OR (95%CI)	*P* value
Age	0.99(0.97,1.01)	0.32	0.95(0.90,1.01)	0.09
Sex	1.51(0.81,2.82)	0.20	1.57(0.42,5.90)	0.50
Diabetes	0.54(0.25,1.16)	0.11	3.40(0.32,36.27)	0.28
Hypertension	0.94(0.40,2.20)	0.88	0.44(0.07,2.76)	0.37
Intraventricular hemorrhage	0.48(0.21,1.08)	0.07	0.18(0.04,0.78)	**0.02**
Blend sign	0.27(0.10,0.78)	**< 0.01**	0.10(0.02,0.49)	**< 0.01**
Island sign	0.17(0.06,0.46)	**< 0.01**	9.89e(-10)	**< 0.01**
Whirl sign	0.43(0.23,0.80)	**< 0.01**	0.20(0.04,0.94)	**0.03**
Fluid level	1.49e(-9)	**0.05**	3.58e(-9)	0.09
Black hole	0.25(0.09,0.72)	**< 0.01**	1.15e(-9)	**0.01**
Hematoma volume	0.96(0.94,0.99)	**< 0.01**	0.93(0.88,0.98)	**< 0.01**
Density grading	0.35(0.24,0.52)	**< 0.01**	0.18(0.05,0.57)	**< 0.01**
Shape grading	0.59(0.47,0.74)	**< 0.01**	0.18(0.06,0.57)	**< 0.01**
Shape-Density Grading*	0.71(0.61,0.82)	**< 0.01**	0.33(0.16,0.69)	**< 0.01**
BH-WS-IS-M*	0.38(0.26,0.55)	**< 0.01**	0.04(0.01,0.36)	**< 0.01**

* Shape-Density Grading, the fusion of density grading and Shape grading; BH-WS-IS-M, the fusion of black hole, whirl sign, island sign and blend sign. P-values are statistically significant in boldface format. OR, Odds ratio. CI, Confidence interval

### Performance of the different models

After training, a total of three distinct interpretable random forest prognostic models were obtained. For the external test set, the Radiomics Model performed with the Area Under the Receiver Operating Characteristic Curve (AUC) of 0.83 (95% CI: 0.67, 0.93; *P* < 0.01), the Radiomics-Clinical Model exhibited an AUC of 0.86 (95% CI: 0.71, 0.95; *P* < 0.01), and the DL-Radiomics-Clinical Model demonstrated an AUC of 0.89 (95% CI: 0.74, 0.97; *P* < 0.01). Further detailed model evaluation metrics can be found in Table 3. The results of the Delong test are presented in Table 4, indicating no statistically significant differences among the ROC curves of the various models.

**Table 3:** Detailed evaluation information of the three models

Datasets	Metrics	Radiomics Model	Radiomics-Clinical Model	DL-Radiomics-Clinical Model
Training cohort	AUC	0.96(95%CI:0.92,0.98; *P* < 0.01)	0.96 (95%CI:0.91,0.98; *P* < 0.01)	0.96(95%CI:0.92,0.99; *P* < 0.01)
	Sensitivity	0.92	0.87	0.93
	Specificity	0.88	0.92	0.85
External test cohort	AUC	0.83(95%CI:0.67,0.93; *P* < 0.01)	0.86(95%CI:0.71,0.95; *P* < 0.01)	0.89 (95%CI:0.74,0.97; *P* < 0.01)
	Sensitivity	0.72	0.78	0.72
	Specificity	0.94	0.89	0.99

AUC, Area Under Receiver Operating Characteristic Curve

**Table 4:** Results of the delong test for the three models

	Radiomics Model	Radiomics-Clinical Model	DL-Radiomics-Clinical Model
**Radiomics Model** (AUC:0.83; SE:0.07; 95%CI: [0.67,0.93])	-	Z statistic:0.72; *P* = 0.47	Z statistic:1.31; *P* = 0.19
**Radiomics-Clinical Model** (AUC:0.86; SE:0.06; 95%CI: [0.71,0.95])	-	-	Z statistic:0.83; *P* = 0.40
**DL-Radiomics-Clinical Model** (AUC:0.89; SE:0.06; 95%CI: [0.74,0.97])	-	-	-

AUC, Area Under Receiver Operating Characteristic Curve. SE, Standard Error of Mean. CI, Confidence interval

**Fig. 4:**
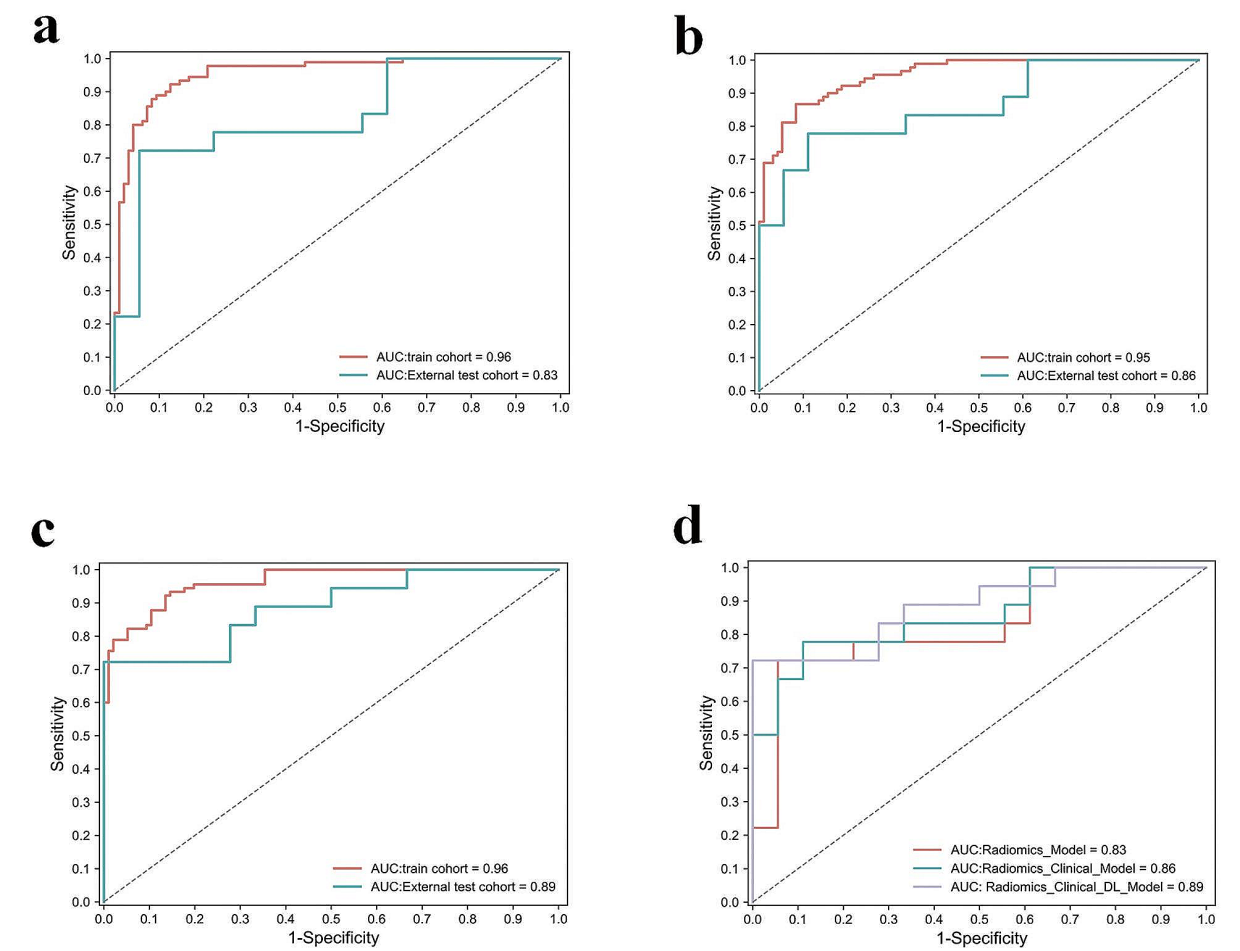
ROC of the models on the training set and external test set. ROC for Radiomics Model (**a**);ROC for Radiomics-Clinical Model (**b**); ROC for DL-Radiomics-Clinical Model (**c**); Comparison of the ROC of the three models on an external test set (**d**)

**Fig. 5:**
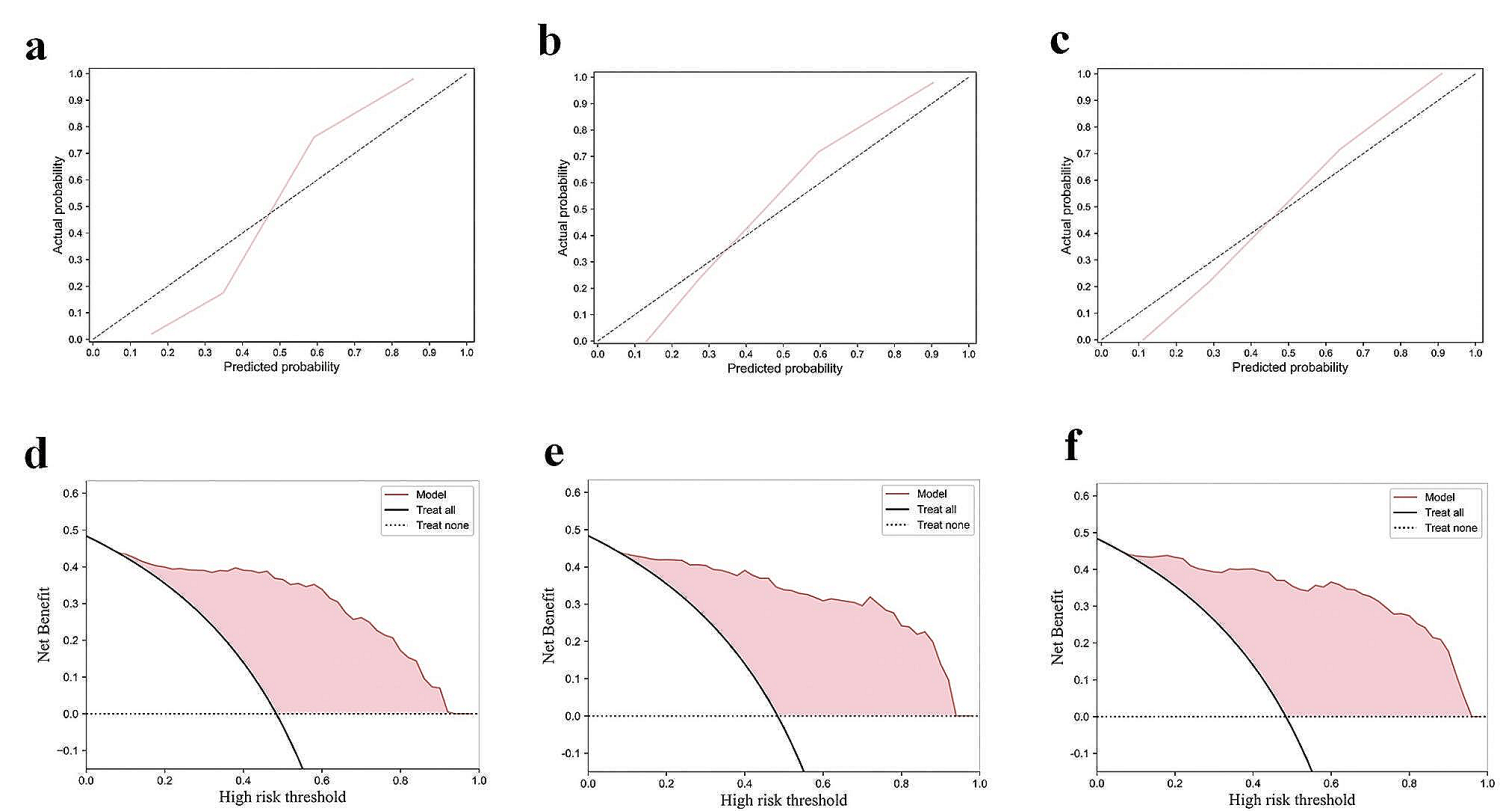
Calibration curves and DCA curves for the three models. Calibration curves for the Radiomics Model (**a**); the Calibration curves for Radiomics-Clinical Model (**b**); Calibration curves for DL-Radiomics-Clinical Model (**c**); DCA curves for the Radiomics Model (**d**); DCA curves for the Radiomics-Clinical Model (**e**); DCA curves for DL-Radiomics-Clinical Model (**f**)

The ROC curves of the Radiomics Model, Radiomics-Clinical Model, and DL-Radiomics-Clinical Model on the training and external test sets are shown in Fig. 4a and b, and Fig. 4c, respectively. A comparison of the ROC curves for the three models on the external test set is illustrated in Fig. 4d. It is evident that the DL-Radiomics-Clinical Model, which incorporates the most modal features, demonstrates the best generalization performance. Following this, the Radiomics-Clinical Model also shows improved predictive capability compared to the single-modal model. This indicates that multimodal features play a crucial role in enhancing the upper limit of the model’s performance. The performance of the calibration curves and Decision Curve Analysis (DCA) curves for the three models is illustrated in Fig. 5. It can be observed from the figure that the two multimodal models exhibit higher linearity, indicating better model calibration effects. From Fig. 5d and e, and Fig. 5f, it can be observed that the Radiomics-Clinical Model and DL-Radiomics-Clinical Model exhibit relatively better clinical utility.

### Research on model interpretability

**Fig. 6:**
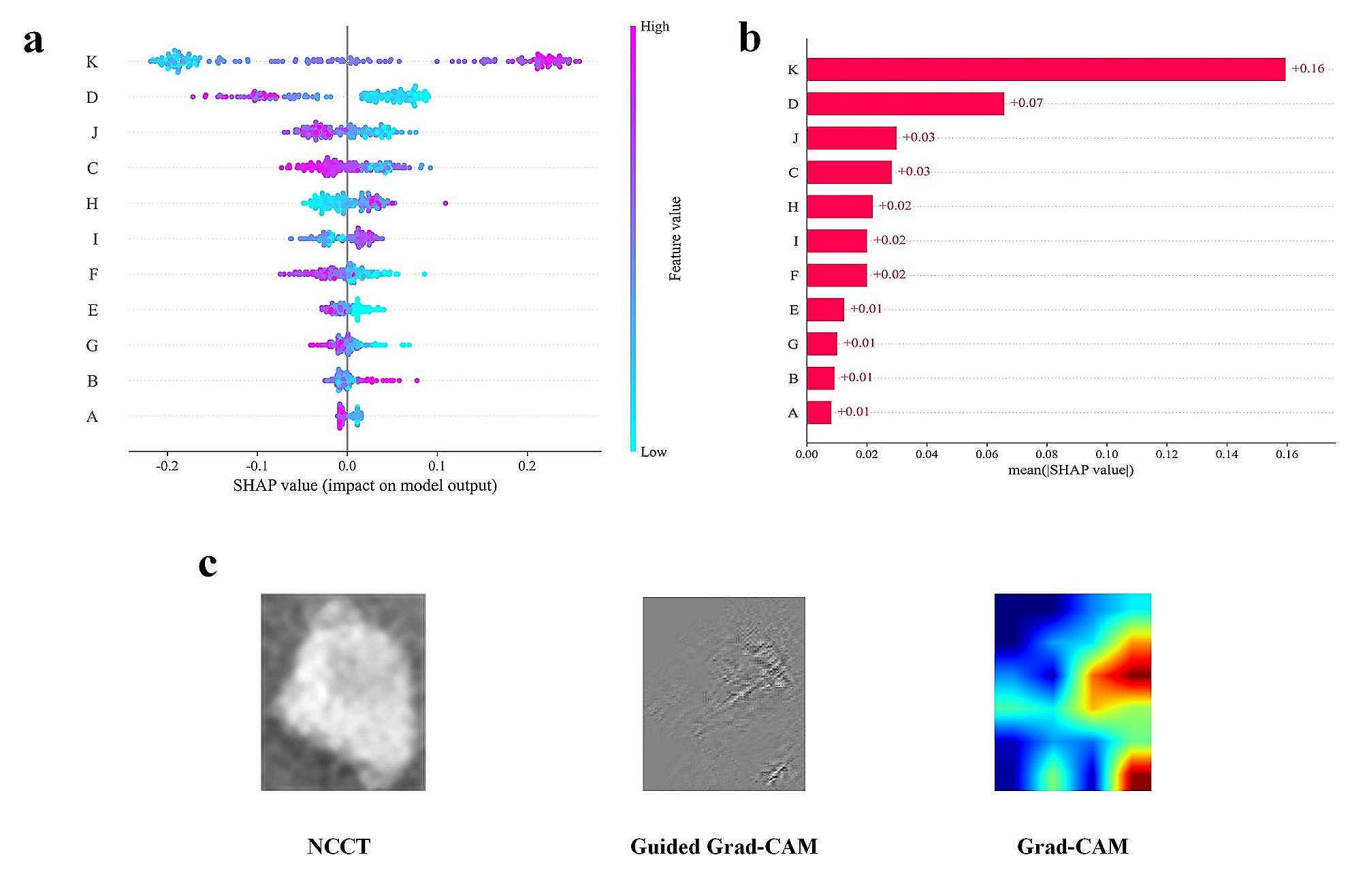
Decision Interpretation for the DL-Radiomics-Clinical Model. SHAP Summary Plot (**a**); The SHAP values for different features(**b**); Model Interpretation Based on Grad-CAM and Guided Grad-CAM (**c**). Where A: ‘Density Grading’, B: ‘wavelet-LHL-firstorder-Skewness’, C: ‘wavelet-LLH-gldm-DependenceEntropy ‘, D:’ log-sigma-2-0-mm-3D-glszm-GrayLevelNonUniformity ‘, E: ' BH-WS-IS-B ‘, F: ' wavelet-LLH-glszm-SmallAreaLowGrayLevelEmphasis ‘, G:‘2D-DL-Features-5’, H:‘log-sigma-4-0-mm-3D-glrlm-RunLengthNonUniformityNormalized’, I:‘original-shape-Sphericity’, J:’ original-shape-LeastAxisLength ‘, K:‘DL-Clinical-Rad-Score’

In order to assess the contribution of each feature to the model predictions, this study utilized SHAP values to decompose and quantify the impact of individual features on the model’s decisions. SHAP is a global interpretability method based on the Shapley value theory from cooperative game theory, enabling the calculation of the importance of each input feature for the model predictions. Figure 6a illustrates the SHAP explanation results of the DL-Radiomics-Clinical Model, where each data point represents a sample. The horizontal axis represents the SHAP value, while the vertical axis represents the features. The position of features on the vertical axis indicates their importance, with higher positions indicating larger contributions. The closer the data points are to the central line, the smaller the contribution of the feature to the predicted outcome, while data points further from the central line indicate a larger contribution of the feature to the prediction. The color of the data points can represent the magnitude of the feature values. According to the results shown in the figure, it can be observed that certain radiomics features related to image grayscale levels and shape exhibit higher model contributions, such as log-sigma-2-0-mm-3D-glszm-GrayLevelNonUniformity, original-shape-LeastAxisLength, and wavelet-LLH-gldm-Dependence Entropy. Figure 6b provides detailed contributions for these features, which are 0.07, 0.03, and 0.03, respectively. Furthermore, the imaging features Density Grading, and BH-WS-IS-M contributed 0.01 each, while the deep learning feature 2D-DL-Features-5 also contributed 0.01. Notably, the fused feature DL-Clinical-Rad-Score made the most significant contribution to the overall model decisions, with a SHAP value of 0.16.

This study introduces a novel fusion feature, DL-Clinical-Rad-Score, through multimodal feature fusion techniques, which captures more intricate relationships among multimodal features. This further assists the model in better leveraging various types of feature information comprehensively. SHAP analysis results also demonstrate that the DL-Clinical-Rad-Score feature contributes significantly. Among the image radiomics features finally included in the training, two first-order features describing lesion shape (describing lesion sphericity and the minimum axis length of 3D shape features) and four features quantifying image grayscale (gray-level size zone matrix features and gray-level dependence matrix) rank highly. These include two features obtained after Gaussian Laplacian operator and two wavelet transform calculations. Generally, the Gaussian Laplacian operator is a two-dimensional isotropic measure of the image’s second derivative, emphasizing areas of rapid intensity changes in the image, primarily used in edge detection tasks. Wavelet transforms analyze the spatiotemporal and frequency limitations, refining the analysis of signals through operations like dilation and translation, effectively extracting information. Gray-level dependence matrices and gray-level size zone matrices, as texture features in radiomics, mainly describe voxel grayscale distribution and variation, with subtle differences aiding in better predicting tumor heterogeneity. Additionally, two deep learning features, Density Grading and BH-WS-IS-B, extract different semantic levels of image features, complementing a broader range of feature information, thereby surpassing and enhancing the model’s performance.

Grad-CAM is a local interpretability method designed for CNN networks. It obtains heatmaps for each pixel by weighting the gradients of the convolutional layer feature maps, providing explanations for the model’s local prediction results. Guided Grad-CAM builds upon Grad-CAM by incorporating guided backpropagation to constrain the influence of non-interpretable regions, resulting in clearer and more precise heatmaps that highlight key feature areas in the model’s decision-making process. Figure 6c illustrates the useful feature information captured by the deep learning model after training, showing the model’s capture of highly heterogeneous regions within the hematoma ROI. More technical details about SHAP, Grad CAM, and Guided Grad CAM are presented in Supplementary material 4.

## Discussion

Compared to ischemic stroke, ICH exhibits more severe neurological symptoms and a higher mortality rate during the acute phase. The majority of ICH cases are attributed to ruptures of arteriole with diameters ranging from 50 to 200 micrometers. [40], Studies have indicated that chronic hypertension may be a significant factor contributing to arteriole ruptures [41]. Following a cerebral hemorrhage event, the primary cause of early neural damage is attributed to increased intracranial pressure, leading to compression and disruption of brain tissue structure [42]. Additionally, a significant factor contributing to neurological dysfunction in ICH is cerebral edema and the mass effect caused by the hematoma leading to compression. With treatment and rehabilitation care, affected tissues can experience a certain degree of recovery [43]. Early identification of the prognosis of ICH can assist clinical physicians in making better clinical decisions and allocating intensive care resources more effectively.

This study incorporates various common imaging features to assist in the construction of a prognostic model, including density grading, black hole, swirl sign, island sign, and blend sign. Barras et al. proposed shape grading and density grading features based on the irregularity of hematoma margins and mixed density. The study demonstrated that mixed density signs and irregularity of margins observed on NCCT were associated with 24-hour hematoma expansion [44]. Research by Eufrozina et al. indicates that the swirl sign can serve as an independent predictive factor for forecasting the prognosis of patients with ICH [10]. Furthermore, in the population with increasing hematoma volume, 39.3% exhibit the blend sign, and among patients showing the blend sign, 82.7% experience early hematoma volume expansion. The sensitivity, specificity, positive predictive value, and negative predictive value of the blend sign for predicting early hematoma volume expansion are 39.3%, 95.5%, 82.7%, and 74.1%, respectively [8]. The black hole feature, identified on NCCT, is a relatively straightforward imaging marker that can independently predict hematoma expansion. Studies have shown that the black hole sign has a specificity of 94.1% for predicting early hematoma expansion [9]. Research by Li et al. demonstrates that the island sign is a reliable imaging marker that can independently predict hematoma expansion and poor prognosis in patients with intracerebral hemorrhage. The island sign on NCCT may serve as a potential marker for therapeutic intervention [7].

The Radiomics Model, Radiomics-Clinical Model, and DL-Radiomics-Clinical Model constructed in this study showed promising performance in predicting the prognosis of patients with intracerebral hemorrhage. However, comparatively, the DL-Radiomics-Clinical Model demonstrated superior performance in all aspects. The DL-Radiomics-Clinical Model utilizes radiomics features, imaging features, and deep learning features as training data, representing image features at different semantic levels. Generally, deep learning features are typically at lower semantic levels, capturing basic patterns and representations within the images. Radiomic features are at a moderate semantic level, providing information about disease morphology and tissue structure. They transform medical images into exploitable data for subsequent analysis, offering decision support [16]. On the other hand, imaging features belong to a higher semantic level, providing more specific and abstract patient anatomical and pathological information. However, they are generally limited to a few qualitative descriptors and subjective observer interpretations [45]. In general, deep learning features, radiomics features, and imaging features capture information at different levels [46]. By integrating knowledge and characteristics from various sources of information, a more comprehensive and accurate feature representation is provided for model training, thereby enhancing the performance ceiling of the model. The DL-Clinical-Rad-Score is a composite feature that combines multimodal features, aiding the model in better understanding the data, as evidenced in Fig. 6b.

In terms of model selection, the RF model is a complex and efficient machine-learning model. However, its interpretability is relatively weak, limiting its applicability in medical tasks [26, 47]. This study employed the SHAP method to explain the decision-making process of the RF model, quantifying the impact of each feature involved in the decision on the model’s outcome. This greatly enhances the interpretability of the RF model, allowing it to balance interpretability while maintaining high performance. For the explanation of the deep learning feature extraction model, this study utilized two visual-based model interpretability tools, Guided Grad-CAM, and Grad-CAM, to reflect the attention of the deep learning model and locate the key regions where the model extracts feature from the images. This study utilized the EfficientNetV2 model for deep learning feature extraction, employed pyradiomics for radiomics feature extraction, and fused them with typical radiological features. Through training a random forest model, we developed a lightweight prognosis model for brain hemorrhage. Due to its lightweight nature, the model has minimal hardware requirements, enabling easy deployment on most current devices.

This study also has some limitations. Although this study incorporates multicenter data for research, adding more center data for training would still benefit the generalization and accuracy improvement of the model, especially for complex artificial intelligence algorithms. The multimodal feature fusion method used in this study is an early fusion approach, which is relatively simple for model construction. However, exploring more advanced multimodal feature fusion models may further enhance the effectiveness of the prognosis model for brain hemorrhage. In addition, Hematoma expansion has been shown to be closely associated with the prognosis and functional impairment of ICH, with over half of ICH patients potentially experiencing hematoma expansion within 24 h of symptom onset [48]. Proactively preventing hematoma expansion is also a potential treatment strategy for ICH. However, due to the limitations of the included data in this study, some patients were missing repeat head CT imaging within 24–48 h after treatment. Including clinical evidence of hematoma expansion in future prospective studies may be beneficial for predicting patient outcomes. Furthermore, in terms of the interpretability of the deep learning model, while the visual interpretability methods of Guided Grad-CAM and Grad-CAM used in this study can provide some visual interpretability, they still cannot quantitatively assess the specific feature impacts like the SHAP analysis method. In future research, we will further investigate additional methods that can provide higher accuracy and interpretability for medical prognosis models.

Explainable medical artificial intelligence emerges as an inevitable direction for the future development of medical AI. With the widespread adoption of new technologies like 5G, the Internet of Things, and wearable sensors, these devices serve as crucial carriers in healthcare. They collect extensive multimodal patient data, allowing users to utilize multimodal predictive models to provide early warnings for high-risk patients based on real-time data processing outcomes.

## Conclusion

This study developed a novel multimodal interpretable artificial intelligence model for predicting the prognosis of brain hemorrhage. In comparison to previous relevant studies [49–51], this study innovatively proposes an interpretable machine learning radiomics framework, integrating three different semantic levels of image features for multimodal model training. This approach enriches data representation, aiding in enhancing both data characterization and model performance limits. Three interpretable model methods, namely SHAP, Grad-CAM, and Guided Grad-CAM, provide quantitative and visual explanations of feature contributions to the prognosis model for brain hemorrhage, facilitating a better understanding of the basis for model decisions. Additionally, this study utilizes multicenter data and external test sets for generalization evaluation, ensuring fair model results and mitigating the occurrence of model overfitting.

### Electronic supplementary material

Below is the link to the electronic supplementary material.

Supplementary Material 1

Supplementary Material 2

Supplementary Material 3

Supplementary Material 4

## Data Availability

The data that support the findings of this study are not openly available due to reasons of sensitivity and are available from the corresponding author upon reasonable request.

## Declarations

### Methodology

Retrospective; Diagnostic or prognostic study; Multicenter study.

### Guarantor

The scientific guarantor of this publication is Chao Yang.

## Abbreviations

AI: Artificial Intelligence
NCCT: Non-contrast Computed Tomography
VOI: Volume of Interest
RF: Random Forest
AUC: Area Under the Receiver Operating Characteristic Curve
DCA: Decision Curve Analysis
SHAP: Shapley Additive exPlanations
CI: Confidence Intervals
ICH: Intracerebral Hemorrhage
GCS: Glasgow Coma Scale
NIHSS: NIH Stroke Scale
CTA: Computed Tomography Angiography
mRS: Modified Rankin Scale
NIFTI: Neuroimaging Informatics Technology Initiative
ROI: Region of Interest
IBSI: Image Biomarker Standardization Initiative
ICC: The Intraclass Correlation Coefficient
mRMR: Minimal-Redundancy-Maximal-Relevance Criterion
MLP: Multi-Layer Perception
SMOTE: Synthetic Minority Oversampling Technique
OR: Odd Ratio
