# Supplementary material for: An interpretable artificial intelligence model based on CT for prognosis of intracerebral hemorrhage: a multicenter study
Source: BMC Med Imaging. 2024 Jul 9;24:170. doi: 10.1186/s12880-024-01352-y (PMC11234657; doi:10.1186/s12880-024-01352-y)
Supplement: Supplementary file 1 — Supplementary Material 1 [file 12880_2024_1352_MOESM1_ESM.docx]

**Supplementary material 1** Details of image protocols and parameters

| **Parameters** | **Center 1** | **Center 2** |
| --- | --- | --- |
| CT version | Philips Ingenuity CT 128-slice, USA or Siemens CT 128-slice, Germany | 128-slice spiral CT (United Imaging, China) |
| CT tube voltage | 120 kVp | 120 kVp |
| CT tube current | 120-550 mA (the duration of automatically optimized to provide similar signal strength) | 300 mA |
| CT rotation time | 0.76-0.80 s | 0.80 s |
| CT detector collimation | 20 mm | 20 mm |
| Image matrix | 512×512 | 512×512 |
| Slice thickness | 5 mm | 5 mm |
