# Supplementary material for: An interpretable artificial intelligence model based on CT for prognosis of intracerebral hemorrhage: a multicenter study
Source: BMC Med Imaging. 2024 Jul 9;24:170. doi: 10.1186/s12880-024-01352-y (PMC11234657; doi:10.1186/s12880-024-01352-y)
Supplement: Supplementary file 2 — Supplementary Material 2 [file 12880_2024_1352_MOESM2_ESM.docx]

**Supplementary material 2** The detailed information of the principles of some data processing methods and models used in the research.

1. **Detailed information on the categories and number of 1762 radiomics features extracted in this study.**

All radiomics features can be classified into the following 14 categories： (1) 14 Shape-based features; (2) 23 Gray Level Co-occurrence Matrix features; (3) 14 Gray Level Dependence Matrix features; (4) 5 Neighboring Gray Tone Difference Matrix features; (5)16 Gray Level Size Zone Matrix features; (6) 18 First Order Statistics features; (7) 736 Wavelet features; (8) 16 Gray Level Run Length Matrix features; (9) 460 Laplacian of Gaussian features; (10) 92 Logarithm features; (11) 92 Square Root features; (12) 92 Square features; (13) 92 Exponential features; (14) 92 Gradient features.

1. **Regarding the training of the EfficientNetV2 model, the following are the detailed hyperparameters and information about the MICCAI dataset:**

EfficientNetV2 is an improved version of the deep neural network model introduced by Google following the original EfficientNet. By incorporating larger model scales and enhanced network architecture designs, it further enhances the balance between computational efficiency and performance. EfficientNetV2 has achieved significant advancements in image classification, object detection, semantic segmentation, and other fields, and has been widely applied in computer vision tasks. Its advantage lies in maintaining high accuracy while achieving greater computational efficiency, making it one of the highly regarded models in the current field of deep learning. For more detailed technical information about the EfficientNetV2 model, please refer to the following paper: https://proceedings.mlr.press/v139/tan21a.html

Hyperparameters: We utilized a range of hyperparameters to train the EfficientNetV2 model, including a learning rate of 0.001, momentum, a batch size of 64, weight decay, and others. We employed the SGD optimizer and implemented a learning rate decay strategy to enhance the convergence speed of the model. Furthermore, we applied different data augmentation methods to expand the dataset, such as flipping, center cropping, and scaling.

MICCAI dataset: Our experiments were pretrained using the MICCAI (Medical Image Computing and Computer Assisted Intervention) dataset. This dataset encompasses various tasks related to medical image processing, including segmentation, classification, and detection. We specifically conducted experiments on one task, which involved classifying the grades of gliomas in BraTS 2018. All BraTS multimodal scans are available as NIfTI files and encompass a) native (T1), b) post-contrast T1-weighted (T1Gd), c) T2-weighted (T2), and d) T2 Fluid Attenuated Inversion Recovery (FLAIR) volumes, acquired with different clinical protocols and various scanners from multiple institutions (n=19). For more detailed information, please refer to: <https://www.med.upenn.edu/sbia/brats2018/data.html>.

Using the BraTS2018 dataset for pre-training, the data preprocessing method remains consistent with the training set for the intracranial hemorrhage prognosis task. The hyperparameters and optimizer configuration used during pre-training are consistent with those mentioned in the previous paragraph. After training for 1000 epochs, we obtain the pre-trained model, then fine-tune it on the training set for the intracranial hemorrhage prognosis task. To improve the upper limit of model performance, we choose to train all parameters without parameter freezing.

1. **The Detailed information** **of the preprocessing steps, segmentation process and feature extraction techniques.**

SimpleITK (https://simpleitk.org/) is a simple and efficient Python library for medical image processing, encapsulating the Insight Segmentation and Registration Toolkit (ITK) to provide users with a simpler and more intuitive interface for handling medical image data. In this project, SimpleITK is used to convert NCCT images of selected patients to the Neuroimaging Informatics Technology Initiative (NIFTI) format, and the spatial resolution of the images is resampled to the same specifications using the nearest-neighbor interpolation algorithm provided in SimpleITK. The intensity of all images is normalized to a range of 0 to 1 using the min-max normalization method, and the window width and level of CT images are uniformly adjusted. These preprocessing operations allow us to remove most of the biases caused by different image acquisition instrument parameters.

In terms of acquiring VOI and the maximum ROI, this study involved two radiologists with years of experience in diagnostic imaging using ITK-Snap software (version: 4.0.1, www.itksnap.org) to manually segment lesions layer by layer along the lesion contour. Upon completion, a 3D lesion VOI is automatically generated. Once all VOIs are segmented, SimpleITK and the numpy algorithm library (https://numpy.org/) are utilized to automatically calculate the volume of hematoma for each patient based on voxel size and number. For all patients' NCCT images and corresponding VOIs, SimpleITK is employed to compute the area of each slice's mask based on voxel spacing and number, retaining only the slice with the largest area in each patient's NCCT image for extracting deep learning features. Finally, two radiologists evaluate the NCCT image features that have been confirmed to impact the prognosis of cerebral hemorrhage, followed by a final assessment by a third senior radiologist.

Pyradiomics (https://pyradiomics.readthedocs.io/en/latest/) is a Python library for medical image feature extraction designed to extract rich quantitative features from medical imaging data for assisting in medical diagnosis, prognosis assessment, and disease research. Pyradiomics offers a comprehensive set of feature extraction tools suitable for processing various medical imaging data types including CT, MRI, among others. In this study, this algorithmic library was utilized to extract 1762 radiomics features from each NCCT image, all of which adhere to the Image Biomarker Standardization Initiative (IBSI) standards[1]. As for the extraction of deep learning features, an efficient deep learning classification model was employed. EfficientNet, proposed by Google, is an efficient convolutional neural network architecture aimed at maintaining accuracy while reducing model complexity and parameter count. It achieves a balance between performance and efficiency by employing compound scaling, optimizing the network's depth, width, and resolution simultaneously[2; 3]. To enhance the model's capability in extracting features from medical imaging data, the model was initially trained using the brain tumor grade classification dataset from MICCAI 2018 BRATS[4], and then fine-tuned using the 186 training set images from this study. Upon completion of model training, other parameters were fixed, and the output nodes of the model's final fully connected layer were modified to 10. Subsequently, each sample's ROI images were processed, and the 10 most predictive deep learning features were outputted.

The extracted image features, radiomics features, and deep learning features mentioned above were combined and subjected to feature selection and feature fusion using the mRMR algorithm and ElasticNet algorithm. The training set labels were balanced using the SMOTE algorithm. Subsequently, the selected features and the computed fused features were inputted into a random forest model. GridSearchCV was employed to train and search for the optimal classification model on the training set. Some of the model training parameters were set as follows: n_estimators=25, min_samples_leaf=1, max_leaf_nodes=8, criterion='gini'.

SHAP (SHapley Additive exPlanations)[5] is a method used to explain predictions made by machine learning models. The core idea of SHAP is to decompose the influence of feature values on model predictions into contributions from each feature value. This allows us to understand the importance of each feature on the final prediction. The calculation of SHAP values considers all possible combinations of each feature to determine the relative importance of each feature on the model output. Using SHAP values can help us: 1. Understand model predictions by explaining the influence of each feature on the model prediction to better understand the model's behavior; 2. Determine feature importance: Identify which features are most critical for the model's predictions; 3. Detect interactions between features; SHAP values can reveal how interactions between features affect the model's prediction results.

1. **Detailed technical description of feature selection in this study**

Prior to training the random forest model, we conducted feature selection and fusion on the extracted features, as described in the Feature Screening and Feature Fusion section of the manuscript. For radiomics features, we initially removed unstable features (ICC < 0.75) based on the ICC coefficients calculated from repeated image segmentation. Subsequently, the remaining features were standardized using Z-Score normalization and redundant features were eliminated using either Pearson or Spearman methods. The fused feature Rad-Score was computed using ElasticNet, and simultaneously, the optimal radiomics features were selected using the mRMR algorithm.

Further, clinical factors were incorporated and subjected to feature selection and fusion with imaging features. Initially, single-factor logistic regression analysis was conducted, selecting variables with P < 0.05 as pre-screened meaningful clinical factors. The selected features from this screening were combined with the radiomics features selected in the previous step, and the fused feature Clinical-Rad-Score was computed using ElasticNet. Simultaneously, the optimal features were obtained using the mRMR algorithm.

Finally, deep learning features were incorporated by combining the optimal radiomics features obtained previously with clinical factors, imaging features, and deep learning features. Through the use of the mRMR algorithm, 10 optimal predictive features were selected. The fused feature DL-Clinical-Rad-Score was computed using ElasticNet. The calculation process of DL-Clinical-Rad-Score is similar to Rad-Score and Clinical-Rad-Score, where the regression coefficients of ElasticNet are multiplied by the feature values, added together, and then combined with a bias coefficient. The key difference lies in the input features used for ElasticNet.

Through the aforementioned steps, we have obtained the 11 most predictive multimodal features. These include clinical factors, imaging features, radiomics features, deep learning features, and the fused feature DL-Clinical-Rad-Score, which integrates these four modalities.

**REFERENCES**

1 Zwanenburg A, Vallières M, Abdalah MA et al (2020) The Image Biomarker Standardization Initiative: Standardized Quantitative Radiomics for High-Throughput Image-based Phenotyping. Radiology 295:328-338

2 Tan M, Le Q (2019) EfficientNet: Rethinking Model Scaling for Convolutional Neural Networks. In: Kamalika C, Ruslan S, (eds) Proceedings of the 36th International Conference on Machine Learning. PMLR, Proceedings of Machine Learning Research, pp 6105--6114

3 Tan M, Le Q (2021) EfficientNetV2: Smaller Models and Faster Training. In: Marina M, Tong Z, (eds) Proceedings of the 38th International Conference on Machine Learning. PMLR, Proceedings of Machine Learning Research, pp 10096--10106

4 Menze BH, Jakab A, Bauer S et al (2015) The Multimodal Brain Tumor Image Segmentation Benchmark (BRATS). IEEE Trans Med Imaging 34:1993-2024

5 Lundberg SM, Lee S-I (2017) A unified approach to interpreting model predictionsProceedings of the 31st International Conference on Neural Information Processing Systems. Curran Associates Inc., Long Beach, California, USA, pp 4768–4777
