# Supplementary material for: An interpretable artificial intelligence model based on CT for prognosis of intracerebral hemorrhage: a multicenter study
Source: BMC Med Imaging. 2024 Jul 9;24:170. doi: 10.1186/s12880-024-01352-y (PMC11234657; doi:10.1186/s12880-024-01352-y)
Supplement: Supplementary file 4 — Supplementary Material 4 [file 12880_2024_1352_MOESM4_ESM.docx]

**Supplementary material 4** Regarding the usage and integration of Grad-CAM and Guided Grad-CAM, the detailed information of mRMR and the technical information and methodological advantages of SMOTE.

1. **The detailed information of Grad-CAM，Guided Grad-CAM and SHAP:**

**Grad-CAM:**

**Usage:**

- 1. First, obtain the model's feature maps through forward propagation.
  2. Next, compute the gradients of a specific class with respect to the last convolutional layer.
  3. Finally, multiply the gradients with the feature maps and take the average to obtain the class activation map (CAM).

**Integration:**

The generated class activation map from Grad-CAM can be overlaid onto the original image for visualizing the model's focus on different regions.

**Guided Grad-CAM:**

**Usage:**

1. Start with the class activation map obtained from Grad-CAM.
2. Utilize backpropagation to retain gradient information in areas with positive gradients while suppressing it in regions with negative gradients.
3. Apply the processed gradients to the original image to obtain the Guided Grad-CAM visualization.

**Integration**:

The results from Guided Grad-CAM can be superimposed onto the original image to provide a clearer representation of the decision-making process of the model.

For more technical details about these two deep learning visualization interpretability methods, please refer to the following paper: DOI: 10.1109/ICCV.2017.74 .

**SHAP:**

SHAP (SHapley Additive exPlanations) is an interpretability method for explaining machine learning model predictions, inspired by the Shapley values from game theory. The SHAP method helps us understand the contribution of each input feature to model predictions, revealing the logic behind model decisions and the importance of factors.

Here are the main characteristics and steps of the SHAP model interpretability method:

1. Shapley Value Theory: Shapley values are a concept from game theory used to measure the contribution of participants to the outcome of cooperative games. In machine learning, Shapley values are used to measure the influence of each feature on model predictions. Specifically, for a given prediction, Shapley values represent the contribution of each feature to that prediction, i.e., how adding or excluding that feature affects the final prediction result.
2. Local Interpretability: The SHAP method provides explanations for individual samples, explaining the model's prediction for each sample. By computing the Shapley values for each feature, we can understand the influence of each feature on the prediction for that sample.
3. Global Interpretability: In addition to local interpretability, the SHAP method also provides insights into the overall model with global interpretability, revealing the importance of features across all samples. By summarizing and analyzing the Shapley values for all samples, we can determine the overall importance of different features for the model.
4. Model Agnostic: The SHAP method is model agnostic, meaning it can be used to explain any machine learning model, including deep learning models, ensemble models, etc. This makes the SHAP method widely applicable and not limited to specific model types.
5. Visualization: The SHAP method offers various visualization tools to intuitively display the influence of each feature on model predictions. Examples include bar plots, scatter plots of SHAP values, etc., which help users better understand the decision logic of the model.

Overall, SHAP is a powerful tool for model interpretability, helping users understand the decision-making process of machine learning models and the importance of features, thereby increasing trust and understanding of model predictions.

**2. Technical information and methodological advantages of SMOTE**

SMOTE (Synthetic Minority Over-sampling Technique) is an oversampling method used to address class imbalance in datasets. Here are the detailed technical aspects and method advantages of SMOTE:

**Technical Details:**

1. **Oversampling Method:**
   - For the minority class samples, SMOTE balances class distribution by generating synthetic samples.
   - Synthetic samples are created by interpolating points between minority class samples in the feature space.
2. **Synthetic Sample Generation:**
   - For each minority class sample, a nearest neighbor from its k nearest neighbors is randomly selected.
   - A new synthetic sample is randomly generated along the line segment between the selected minority class sample and its neighbor.
3. **Balancing Samples:**
   - The generated synthetic samples increase the number of minority class samples, achieving class balance.

**Method Advantages:**

1. **Mitigating Class Imbalance:**
   - SMOTE effectively mitigates class imbalance issues, improving the model's ability to recognize minority class samples.
2. **Reducing Overfitting Risk:**
   - By adding synthetic samples instead of simply replicating existing minority class samples, the risk of overfitting is reduced.
3. **Improving Classification Performance:**
   - With a balanced dataset, the model can more easily learn the features of minority class samples, thus improving classification performance.
4. **Preserving Data Features:**
   - The generated synthetic samples are interpolated based on the features of existing minority class samples, helping to preserve the data's feature information.
5. **Wide Applicability:**
   - SMOTE is a widely applicable and easy-to-implement oversampling method that finds extensive use across various domains and models.

In summary, SMOTE addresses class imbalance by generating synthetic samples, offering advantages such as improved classification performance, reduced overfitting risk, and preservation of data features. It stands as an effective method for handling class imbalance issues.

**3.The detailed information of mRMR.**

The mRMR method comprises two key concepts:

1. Maximum Relevance: Selecting features highly correlated with the target variable to ensure a significant association between the chosen features and the target variable.

2. Minimum Redundancy: Simultaneously ensuring minimal correlation among the selected features, avoiding the selection of highly correlated features to reduce redundancy.

The mRMR method typically achieves this by computing the correlation between features and the target variable, as well as the correlation among features. Specifically, it first calculates the correlation between each feature and the target variable (often using metrics like Pearson correlation coefficient or information gain), then computes the correlation between each pair of features. Subsequently, it adds the feature with maximum relevance to the already selected feature set while considering its correlation with the previously selected features to minimize redundancy. This iterative process continues until the desired number of features is selected.

The mRMR method finds widespread application in feature selection, particularly in high-dimensional datasets and fields such as bioinformatics. It aids in reducing feature dimensions, improving model generalization, and identifying the most discriminative features, thereby facilitating the understanding of the dataset's structure and relationships between features.
